# Comparison between the Physiological Responses and Subjective Ratings of a Group of Male Students to Three Backpack Designs

**DOI:** 10.3390/ijerph16214104

**Published:** 2019-10-24

**Authors:** Mohamed Z. Ramadan, Sultan N. Al-Tayyar

**Affiliations:** Industrial Engineering Department, Faculty of Engineering, King Saud University, P.O. Box 800, Riyadh 11421, Saudi Arabia; snsst@yahoo.com

**Keywords:** load carriage, treadmill, walking, children, low back pain

## Abstract

It is important for schoolchildren and their parents (or guardians) to know which backpacks exert the least strain on the cardiorespiratory system. In this study, we investigated the physiological responses of participants while they were walking on a treadmill and wearing one of three different backpacks (A, B, and C) under two different load-carrying conditions (equivalent to 10% and 15% of their body mass, respectively). The first condition was used as a control and involved walking without a backpack, while the second involved wearing a backpack and carrying a certain weight. Thirty-one male students from King Saud University walked on a treadmill at 0.861 m/s and at a 0% inclination angle, while having their heart rates (beatsmin^−1^), oxygen uptakes (VO2, mLmin^−1^), respiratory rates (breaths, VO2min^−1^), perceived exertion rates (PER, Borg scale), and backpack preference rates (BPR) measured and recorded. The results of our within-subject experimental design revealed that the physiological results varied significantly depending on the type of backpack. Backpacks B and C were superior to Backpack A, resulting in lower physiological responses and higher subjective preferences. Students carrying more weight experienced higher physiological stress; moreover, the use of Backpack C led to the lowest physiological strains and higher subjective preferences.

## 1. Introduction

Students use schoolbags daily to carry educational material (e.g., heavy books) over long distances. Schoolbags are useful for this purpose; however, if improperly worn or overloaded, they may cause pain or injury to the user [1]. The vast majority of the students who participated to the study described in [2] were regular school bag users; the results of that study were consistent with those of other researchers [3]. Unfortunately, students often carry school bag weights that exceed the limits recommended by several societies [4,5]; for example, more than of 92% of school children in the United States wear schoolbags whose weight exceeds 20% of their body mass [6]. In [7], healthy adults carrying a 12 kg backpack for a short time experienced a significant reduction in the upper extremity perception and in all the macrovascular and microvascular hemodynamic parameters. The need to improve education efficacy for a better society has been previously emphasized [8], and reducing the physical stress arising from the daily transport of heavy backpacks can provide a considerable contribution. 

The incidence of lower back pain among young students (e.g., school-going children) is high, varying between 30% and 70%, depending on the type of pain, age, and research study design [9]. Health and safety professionals, school administrators, and parents have pointed out that regularly wearing school bags is a potential risk factor for lower back pain in children and adolescents [10]. Despite the absence of exact reference values for the mass of school bags, heavy loads have been identified as one of the main causes of back pain [11]. Therefore, most researchers and health practitioners recommend the maximum weight of a schoolbag to be 10% of the body mass of healthy students, and the load to be equally distributed over both shoulders [12,13]. 

Previous research involving children has proved that backpacks weighing between 10% and 30% of the user’s body mass cause a significant increase in the contact pressures beneath the backpack shoulder straps [14]. This pressure exceeds the threshold (30 mm Hg) over which skin blood flow is prevented [15]. Schoolbag straps often put pressure on the anterior part of the shoulder, over the brachial plexus, axillary artery, and vein [16], affecting hand/arm circulation and sensation.

Several researchers have demonstrated that carrying a heavy backpack results in decreased walking speed [17] as well as in increased walking time [18], trunk forward lean [19,20], cardiorespiratory responses [21], loads on the lumbar intervertebral discs [22], and foot-ground pressure [23]. A decrease in the walking speed and an increase in the walking time can be caused by a combination of shorter steps (reduced stride length), fewer steps per minute, and the tendency of spending more time on both feet, rather than just on one foot. For example, college students wearing schoolbags weighting between 16% and 20% of their body mass have been found to spend a longer time walking over a certain distance, compared to students who did not wear such schoolbags [24]. 

The implementation of gait screenings at schools may be useful for the detection of critical conditions exacerbated by load transfer (e.g., obesity, abnormalities in the foot function, and morphology) that may cause discomfort, pain, or decrease the desire of participating in physical activities [25]. Significant postural sways have been observed in healthy adolescents wearing schoolbags weighting 15% of their body mass [26]. Nevertheless, the recommended load limit for school bags should be lessened for age- and sex-matched adolescent idiopathic scoliosis participants, for whom a load equivalent of 10% of their body mass is already sufficient to change their postural stability and bodily direction, and may decrease their endurance to disturbance before a fall.

Some school bag designs focus on the harness [27], which is responsible for conveying a significant portion of the weight to the pelvis. The muscle force necessary for the load transport can be decreased by conveying more of it directly to the pelvis [28]; for instance, ~1/3 of the vertical load can be transferred to the hips by adopting a framed schoolbag with a hip belt [29]. Schoolbags lacking tight hip belts impose additional stress on the shoulders of the subject through the straps. By using a flexible frame, the carried weight can be transferred directly to the pelvis; this is advantageous, since the waist skin is several times less sensitive to pressure than that of the shoulders [30]. By adding lateral stiffness bars to the sides of the schoolbag, it is possible to convey part of the vertical load from the back and shoulders to the waist, decreasing the vertical load supported by the pelvis [31]. To the best of the authors’ knowledge, few studies have investigated the effect of distributing loads around the upper body. In this study, we compared three different backpacks, two of which were designed to distribute the carried loads (e.g., 10% and 15% of the subjects’ body mass) around the upper body.

## 2. Materials and Methods

### 2.1. Participants

Thirty-one college students from King Saud University volunteered for this study; their characteristics are shown in Table 1. These college students were the only ones approved by the Institutional Review Board (IRB), since they had the necessary health insurance (stipulated with the University). In addition, the new design should be implemented on students who have completed their musculoskeletal system.

**Figure 1:**
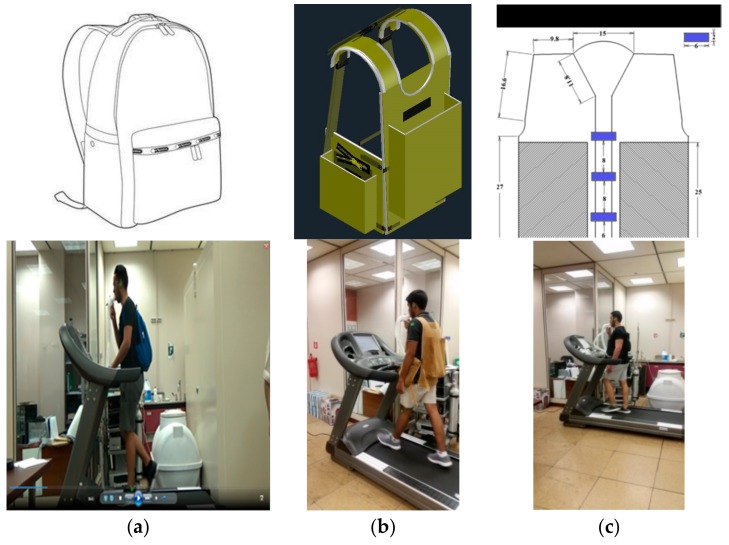
Backpacks used in the experiment. (**a**) popular school backpack; (**b**) and (**c**) backpack fabricated for the experiment.

An informed consent form, approved by the Institutional Review Board of the King Saud University and the College of Medicine (IRB Approval on Research Project No. E-18-3451) was obtained from each student before the start of the experiment. All procedures and methods were performed in accordance with the guidelines and regulations mentioned in the informed consent form and approved by the IRB. In addition, a specific consent form was obtained from each participant for the publication of their photographs on online open-access publications (Figure 1). Afterward, the age, body mass, and height of the students were measured and recorded. The body mass and height were measured using DETECTO’s solo, an eye-level clinical scale (Webb City, Jasper County, Missouri, USA). Despite our repeated attempts at also recruiting female students by distributing leaflets in the girls’ section, only male participants volunteered for the experiment.

### 2.2. Backpacks

**Figure 2:**
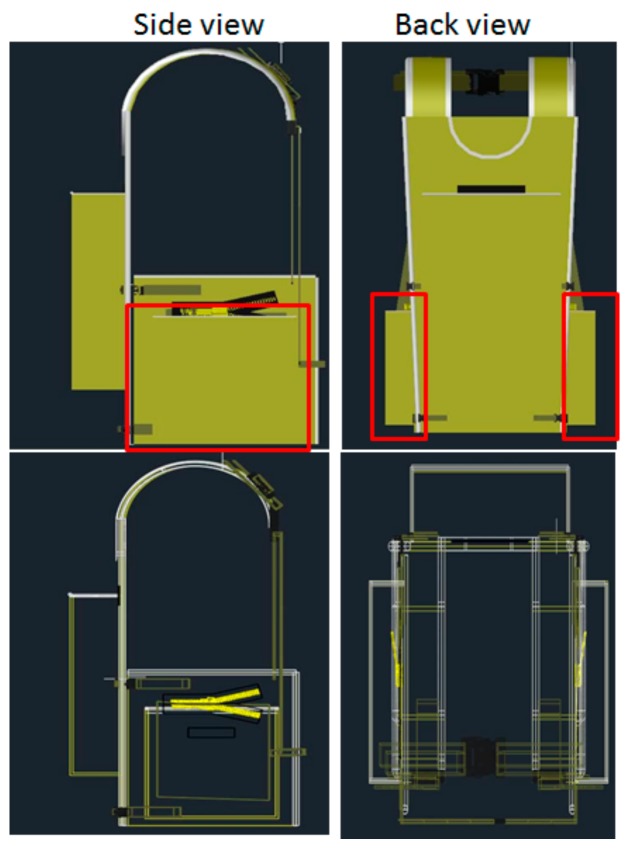
Details of Backpack B.

Backpack A is a popular school backpack, available in the local market, while the other two (B and C) were fabricated for the experiment. Backpack B was similar to the model used in [32] (the Back-T-pack with compression straps), while Backpack C was similar to that used in [13] and included two main compartments (one on the front and one on the back). Backpack B had a back pocket and two side pockets (Figure 2). These three pockets were included since they would help distributing the weight from the back to other areas. The backpacks were filled with sandbags and books until reaching 10% or 15% of the participant’s body mass.

### 2.3. Equipment

A Polar monitor was used to check the participant’s heart rate (Polar of Kemple, Finland); the instrument was covered with a conductive gel and positioned over the subject’s sternum, on clean naked skin. A Moxus Modular Metabolic System using legendary CD-3A and S-3A Gas Analyzers (AEI Technologies, Inc., Bastrop, TX, USA) was used to measure the oxygen uptake and the respiratory rate. The main components of the Moxus Metabolic System included an oxygen analyzer (S-3A/I), a carbon dioxide analyzer (CD-3A), a flow control pump (R-1), a 4.2-L active mixing chamber, a canopy Pump, a canopy Hood, calibration and reference gases, a laptop with an interface software (to calibrate and collect data for further analysis), and a mask worn by the participants (tested for leakage). The mask was connected to the main unit by two flexible pipes. A computer, loaded with software for the recording and analysis of respiratory and heart data, was also connected to the main unit. While the participants were carrying the backpack and walking on a treadmill (OAC297-OLYMPIA; Olympia Fitness Systems, Gujarat, India) [33], their heart rate, oxygen uptake, and respiratory rate were measured and recorded every 10 s.

### 2.4. Experimental Design

The backpack type and the load carried were the independent variables of this study. The heart rate (beats∙min^−1^), VO2 (L·min^−1^ and mL.kg^−1^∙min^−1^), respiratory rate (breaths∙min^−1^), perceived exertion rate (PER), and backpack preference rating (BPR) represented the dependent variables. The PER was measured referring to the Borg scale, considering values between 6 (“no exertion at all”) and 20 (“maximal exertion”). The PER has proved its high reliability in different studies [34,35,36,37,38,39].The BPR was measured referring to values between 0 (“not preferred at all”) and 9 (“very preferred”, which corresponded to the feeling of walking without any load). A within-subject design was adopted for the experiment: the two loads were randomly assigned to each participant wearing the three backpacks one after the other. To minimize the fatigue experienced by the participants, only three loaded backpack runs per day were allowed for each of them. A total of seven trials (2 load levels × 3 backpacks + control (“no-load”)) were executed for each participant. When significant effects were detected, a series of post hoc tests (i.e., LSD tests) were implemented to differentiate between factor levels. For the perceived exertion and backpack preference rating data only, we performed a nonparametric test (i.e., Friedman test); if we observed significant response variables from this data, we then conducted a series of post hoc tests (i.e., Wilcoxon signed-rank tests). All the statistical analyses were performed using the Statistical Package for the Social Sciences (SPSS, Inc., Chicago, IL, USA) version 23.

### 2.5. Experimental Protocol

Before the start of the experiment, all the participants were informed about its purpose and signed consent forms approved by the University Internal Review Board. Then, they were asked to determine the treadmill speed at which they could walk most comfortably while carrying an unloaded backpack. The average speed (3.1 km·hr^−1^) was calculated from those selected by the 31 participants (between 2.9–3.4 km·hr^−1^) and used during all sessions. The participants were then weighed, their heights measured, the resting heart rate read, and their ages recorded for inclusion in the energy measuring program. The participants were then given an orientation and requested to report any feelings of discomfort, which were resolved before continuing the testing. The backpack’s bottoms were adjusted at the level of each participant’s waist.

The participants were fitted with a nosepiece for the collection of expiratory air, and a Moxus Modular metabolic system measured the VO2 consumption during the walking procedures. Each of these procedures was conducted for ~5 min in order to attain a “steady-state”, ideal for the collection of metabolic data. The participants were asked to rest on a chair until their heart rates normalized or were within 5 beats·min^−1^, before conducting a new test [13]. The PER and BPR were measured and recorded at the end of the walking session. 

The testing of each participant was completed in ~2 h, while the overall data collection lasted over 3 weeks. A free-load test was performed at the beginning of the experiment, followed by the random testing of six different conditions (using three backpacks and two different loads). During the testing sessions, the participants were dressed in shorts, t-shirt, socks, and sports shoes; additionally, each participant came into the lab at the same time of the day. The test was conducted during the spring semester, at a mean room temperature of 21–22 °C and relative humidity of 50%–60%. The participants were instructed not to eat anything and to avoid exercise and caffeine for at least 4 h beforehand [40]. 

## 3. Results

The results of the reliability analysis, based on the Cronbach coefficient (α), were used to calculate the internal consistency coefficients of the subjective factors, which were satisfactory: 0.836 and 0.751 for the backpack preference and perceived exertion ratings, respectively. Negative linear regression coefficient (p < 0.0001) was found between RPE and BPR with Spearman correlation (−0.915).

### 3.1. Heart Rate

**Figure 3:**
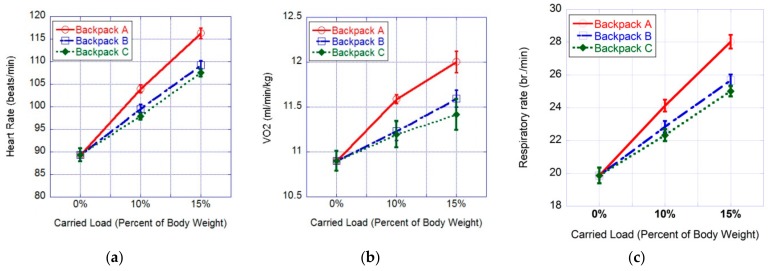
Effects of different backpacks, represented by the percent of carried load interaction with respect to various physiological responses. (**a**) Heart rate response; (**b**) Oxygen uptake response; (**c**) Respiratory rate response.

Our results showed that the heart rate was significantly affected by the backpack type (F (2,60) = 52.373, *p* < 0.0001, η^2^ = 0.636), the percent of carried load with respect to the participant’s body mass (F (2,60) = 111.872, *p* < 0.0001, η^2^ = 0.789), and the percent of carried load interaction (F (4,120) = 15.866, *p* < 0.0001, η^2^ = 0.346). When the carried load corresponded to 10% of the participant’s body mass, the LSD tests revealed that the participant’s heart rate was significantly higher while wearing Backpack A (p < 0.002), rather than Backpacks B and C (*p* < 0.0001); meanwhile, no significant differences were found for this parameter between Backpacks B and C. When the carried load corresponded to 15% of the participant’s body mass, the LSD tests revealed that the participant’s heart rate was significantly higher in the case of Backpack A (*p* < 0.0001) compared to Backpacks B and C (p < 0.0001); meanwhile, no significant differences were found between Backpacks B and C (Figure 3a).

### 3.2. VO2·kg^−1^

Our results showed that the VO2·kg^−1^ was significantly affected by the backpack type (F (2,60) = 7.313, *p* < 0.001, η^2^ = 0.196), the percent of carried load (F (2,60) = 17.027, *p* < 0.0001, η^2^ = 0.362), and the percent of carried load interaction (F (4,120) = 2.616, *p* < 0.039, η^2^ = 0.08). When the carried load corresponded to 10% of the participant’s body mass, the LSD tests revealed that the participant’s VO2·kg^−1^ was significantly higher when using Backpack A (*p* < 0.029), rather than Backpacks B and C (*p* < 0.016); however, no significant differences were found between Backpacks B and C. When the carried load corresponded to 15% of the participant’s body mass, the LSD tests revealed that the participant’s VO2·kg^−1^ was significantly higher in the case of Backpack A (*p* < 0.034) compared to backpacks B and C (*p* < 0.003); meanwhile, no significant differences were found between Backpacks B and C (Figure 3b).

### 3.3. Respiratory Rate

Our results showed that the respiratory rate was significantly affected by the backpack type (F (2,60) = 28.889, *p* < 0.0001, η^2^ = 0.491), the percent of carried load (F (2,60) = 137.363, *p* < 0.0001, η^2^ = 0.821), and the percent of carried load interaction (F (4,120) = 14.88, *p* < 0.0001, and η^2^ = 0.332). When the carried load corresponded to 10% of the participant’s body mass, the LSD tests revealed that the participant’s respiratory rates were significantly higher when using Backpack A (*p* < 0.013), rather than Backpacks B and C (*p* < 0.001); however, no significant differences were found between Backpacks B and C. When the carried load corresponded to 15% of the participant’s body mass, the LSD tests revealed that the participant’s respiratory rates were significantly higher in the case of Backpack A (*p* < 0.0001) compared to backpacks B and C (*p* < 0.0001); meanwhile, no significant differences were found between Backpacks B and C (Figure 3c).

### 3.4. Perceived Exertion Rating

**Figure 4:**
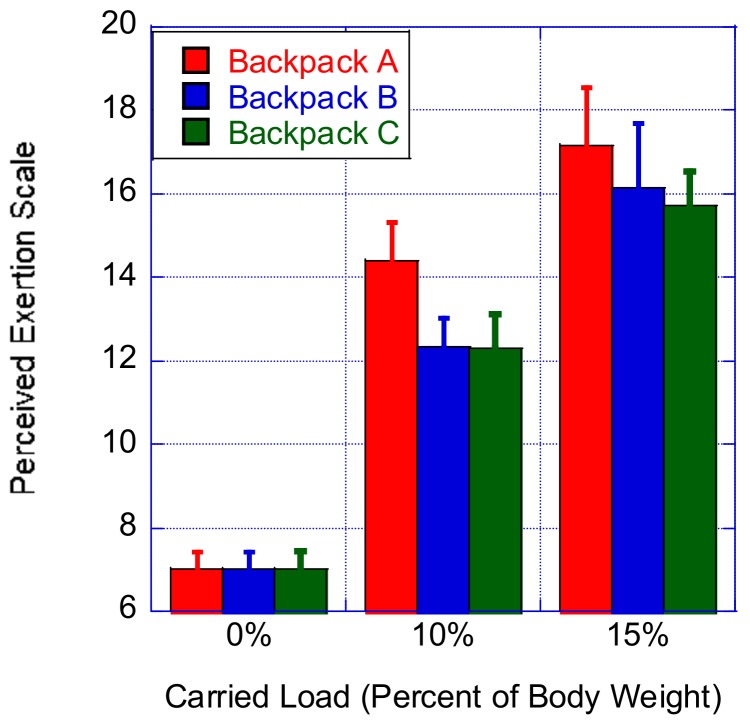
Effect of the percent of carried load on the perceived exertion.

The results of the Friedman test showed that there were no statistically significant differences in the perceived exertion depending on the backpack type. The following statistical values were obtained for loads corresponding to 10% and 15% of the body mass, respectively: χ^2^(2) = 60.612, *p* < 0.0001; χ^2^(2) = 62.00, *p* < 0.0001. When the carried load corresponded to 10% of the participant’s body mass, a post hoc analysis (i.e., Wilcoxon signed-rank tests) was conducted, and a Bonferroni correction was applied. We noted a significant reduction of the perceived exertion of Backpack A compared to either Backpack B (Z = −4.78, *p* < 0.0001) or Backpack C (Z = −4.65, *p* < 0.0001); meanwhile, no significant differences were found between Backpacks B and C concerning the perceived exertion. When the carried load corresponded to 15% of the participant’s body mass, another post hoc analysis (i.e., Wilcoxon signed-rank tests) was conducted, and a Bonferroni correction was applied. We noted a significant reduction of the perceived exertion of Backpack A compared to either Backpack B (Z = −2.61, *p* < 0.009) or Backpack C (Z = −3.889, *p* < 0.0001) (Figure 4). Meanwhile, no significant differences were found between Backpacks B and C concerning the perceived exertion.

### 3.5. Backpack Preference Rating

**Figure 5:**
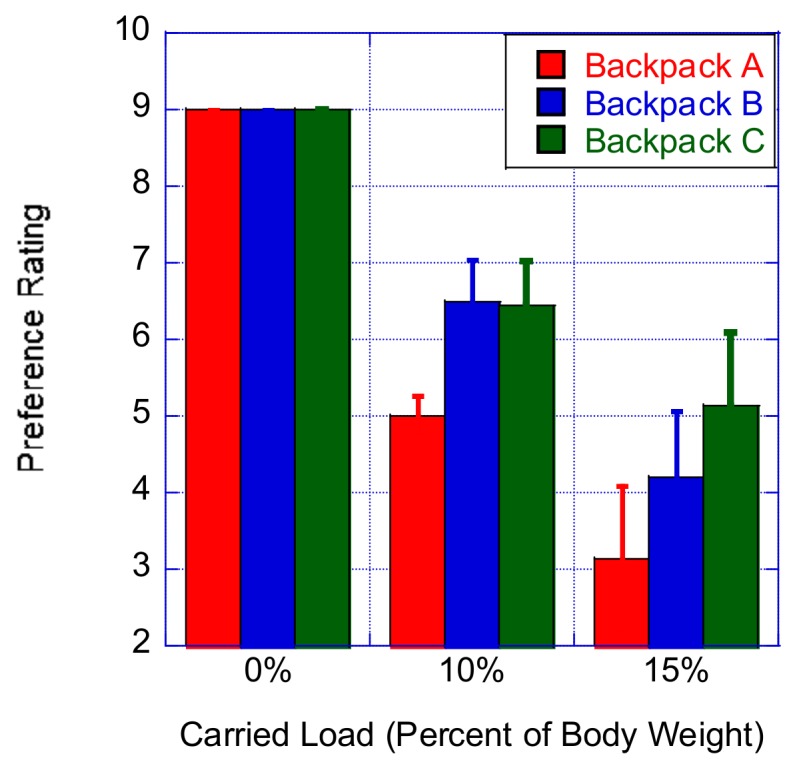
Effect of the percent of carried load on the backpack preference rating.

The results of the Friedman test showed that there were statistically significant differences in backpack preference rating depending on the backpack type worn when carrying loads corresponding to 10% (χ^2^(2) = 50.596, *p* < 0.0001) or 15% of the body mass (χ^2^(2) = 43.676, *p* < 0.0001). When the carried load corresponded to 10% of the participant’s body mass, a post hoc analysis (i.e., Wilcoxon signed-rank tests) was conducted and a Bonferroni correction was applied. We noted a significant reduction of the backpack preference rating of Backpack A compared to either Backpack B (Z = −5.011, *p* < 0.0001) ) or Backpack C (Z = −5.015, *p* < 0.0001); meanwhile, no significant differences were found between Backpacks B and C concerning the backpack preference rating. When the carried load corresponded to 15% of the participant’s body mass, another post hoc analysis (i.e., Wilcoxon signed-rank tests) was conducted, and a Bonferroni correction was applied. We noted a significant increase of the backpack preference rating of Backpack A compared to either Backpack B (Z = −3.979, *p* < 0.0001) or Backpack C (Z = −4.679, *p* < 0.0001) (Figure 5). In addition, the participants expressed a significantly higher preference for Backpack C compared to Backpack B (Z = −3.556, *p* < 0.0001) (Figure 5).

## 4. Discussion

The purpose of this study was to compare the effects of three different backpack designs on the physiological responses and subject measurements of a group of students. Backpack C distributed the carried load on the back (50% of the load) and on the sides (25% of the load on each side), Backpack B distributed it on the back and front (50% of the load on each side), and Backpack A concentrated the carried load only on the back. The results of this study showed the occurrence of significant differences in the physiological responses and backpack preference measurements of students to the three backpack designs. Fewer physiological responses (i.e., the lowest heart rate, respiration rate, and oxygen uptake values) were associated with wearing Backpack C. Furthermore, the findings in this study are consistent with those of previous researches [41], demonstrating that increasing carried loads increase both the metabolic costs associated with walking and the pressure under the feet [42].

In our study, the students preferred using Backpack C, especially when carrying loads equivalent to 15% of their body mass; meanwhile, the subjective ratings reported in [43] were not sufficient for identifying any subjective perceptual differences in relation to different backpacks. In fact, the backpacks compared in that study were very similar to each other (i.e., presented only slight variations in the design features), while those compared in our study were considerably different. These data suggest that subjective ratings can be used efficiently for the analysis of distinct backpack designs. It is not surprising that subjective participant perceptions of ease of use were affected while wearing Backpack C. The student’s subjective ratings were in line with the perceived exertion ratings [44]. 

Further research is required to investigate any variations in the effects of carried loads and backpack designs on both genders, as well as in relation to the transport of backpacks over different periods (e.g., while adopting different postures). A similar study considering a larger sample size may reveal significant differences between distinct school-age groups. Moreover, long-term studies are necessary for determining the long-term effect of wearing backpacks and the load-bearing effect; these would support the establishment of the guidelines for the protection of students from injuries and altered posture.

## 5. Conclusions

The results of this study indicated significant differences in the physiological responses of a group of students to different backpack designs: Backpacks B and C resulted in a significant reduction of strains on the cardiac and respiratory systems. Additionally, the participants showed a higher preference for Backpacks B and C while carrying loads. Therefore, in order to reduce the negative effects of carrying loads for an extended period of time, we recommend the adoption of these new backpack designs. The results of this study further indicated that, when students carried loaded backpacks exceeding 10% of their body mass, their cardiorespiratory system was significantly more strained, especially while wearing the commercial backpack (A); meanwhile, the same load did not have any adverse effects on the participants while they were wearing the modified backpacks (B and C).

The carried weight data of this study were collected in a laboratory environment and, therefore, implied systematic methodological limitations. One of these limitations is that only a limited number of participants could be employed in the experiment; the other is that only male participants were recruited in this study. A prospective study including female participants may elucidate the occurrence of any gender-based differences. Finally, a longitudinal study evaluating the long-term effects of the modified backpacks (B and C) on the cardiorespiratory system could help in identifying the correspondent benefits.

## Figures and Tables

**Table 1:** Characteristics of the participants (N = 31 males).

Variable of Interest	Min	Mean	Max	Standard Deviation
Age (years)	20	25.9	30	2.69
Body mass (kg)	62	75.1	94	8.35
Height (m)	1.56	1.68	1.80	0.05
Body mass index (kg/m^2^)	18.4	25.1	32.53	3.03
